# Cortisol awake response imbalance as an indicator of acute central serous chorioretinopathy: Relationship with choriocapillaris and choroidal features

**DOI:** 10.3389/fmed.2022.1030352

**Published:** 2022-11-30

**Authors:** Fabio Scarinci, Francesca Romana Patacchioli, Eliana Costanzo, Mariacristina Parravano

**Affiliations:** IRCCS – Fondazione Bietti, Rome, Italy

**Keywords:** central serous chorioretinopathy (CSC), salivary cortisol awake response, choroidal vascularity index (CVI), subfoveal choroidal thickness, flow signal void area, optical coherence tomography (OCT), optical coherence tomography angiography (OCTA)

## Abstract

**Purpose:**

The purpose of the present study was to measure in central serous chorioretinopathy (CSC) the salivary cortisol awake response (CAR) delta percentage (Δ%) variation, a distinct and robust indicator of cortisol rhythm during wakefulness, commonly proposed as a marker of hypothalamic-pituitary adrenal (HPA) axis activity, whose alteration is frequently associated with several adverse health outcomes.

**Methods:**

In the present cross-sectional observational study, salivary CAR Δ% variation was assessed in 17 adult male subjects affected by acute naïve CSC and compared to 17 matched healthy controls. Choroid vasculature metrics were assessed in the study population by measuring the subfoveal choroidal thickness (FCT) and the choroidal vascularity index (CVI) by the imaging technique of enhanced-depth imaging spectral-domain optical coherence tomography (EDI-SD-OCT). Furthermore, flow signal void area features of the choriocapillaris were evaluated in the study population using OCT angiography (OCTA).

**Results:**

Both the control and CSC groups showed a physiological cortisol increase that occurred during the first 30 min after awaking. However, CSC adult male patients showed remarkably blunted CAR Δ% variation in comparison with controls, which might reflect a CSC-related imbalance of HPA axis activity. Statistically significant correlations were shown by Pearson’s correlation test between salivary CAR Δ% and the selected choroidal and choriocapillaris imaging biomarkers (FCT, CVI, and flow signal void area) in the study population.

**Conclusion:**

In conclusion, alterations of the CAR Δ% increase, associated with choroidal-retinal metrics, might provide a window into the physiopathology of acute CSC, suggesting a possible common factor to explain the association between stress and CSC.

## Introduction

Central serous chorioretinopathy (CSC) is characterized by thickening of the choroid and abnormal choroidal circulation, which lead to impairment of the retinal pigment epithelium (1). CSC occurs most frequently in midlife and is more common in men than in women (2, 3). Among the several potential causes CSC-related, exposure to high levels of endogenous or exogenous glucocorticoids is considered one of the most important (4, 5). Furthermore, it has been shown that CSC patients have high daily cortisol levels, which reflect hypothalamus-pituitary-adrenal (HPA) axis hyperactivity (5–8).

The links between dysfunction of the HPA axis leading to alterations in diurnal salivary cortisol production and health consequences have already been studied for various medical conditions, including hypertension, burnout, emotional stress, nocturnal apnea, and eating behavior (9–16).

However, the mechanisms by which stress hormone overactivity and allostatic overload could lead to the choroidal hyperpermeability seen in CSC still warrant further investigation (5, 17–19).

A distinct and robust component, an indicator of HPA axis activity, is represented by the characteristic “rhythm within the rhythm,” under the control of the suprachiasmatic nucleus, the principal circadian clock of the brain, namely, the so-called cortisol awakening response (CAR), a post-wakening surge of cortisol that occurs approximately 30 min after awakening (20–24).

The CAR is a complex phenomenon that can be expressed by the calculation of the area under the curve with reference to ground (AUC_*G*_), representing the total level and/or duration of cortisol production at awakening and 30–60 min afterward (20). In contrast, in the present study, we calculated the changes in the increase in cortisol secretion 30 min after awakening, accurately representing a dynamic measure of the shape of the phenomenon (21, 25). Therefore, in this study, salivary CAR Δ% variation 30 min after awakening was calculated by using salivary samples collected at awakening and 30 min later (21, 26) in 17 adult male subjects affected by naïve acute CSC in comparison with 17 matched healthy controls.

The status of choroidal and choriocapillaris microvasculature was assessed qualitatively and quantitatively in the study population using new technologies such as optical coherence tomography (OCT) with the enhanced-depth imaging (EDI) technique, swept-source (SS) OCT, and OCT angiography (OCTA) (19, 27, 28). The choroidal vasculature metrics were assessed by measuring the subfoveal choroidal thickness (FCT) and the choroidal vascularity index (CVI), in healthy controls and CSC patients (29). Furthermore, to gather information on the choriocapillaris status, flow-signal void area features were also evaluated in the study population using OCTA (30).

Finally, we explored the interdependencies among the salivary CAR and all three selected markers from the CSC imaging features (FCT, CVI, and flow signal void area). Therefore, Pearson’s correlation test was used to show any correlations between variables.

## Materials and methods

### Study population

This study was approved by obtained from the Central Ethics Committee for Lazio, Italy (protocol n° 4327/18 April 2018). In the *a priori* sample size calculation, it was estimated that at least 30 subjects (15 per group) were necessary to identify a difference of approximately 30% for the predictable changes in salivary cortisol production 30 min after awakening with α = 0.01, β = 0.2 and a statistical power of 80% (31).

Seventeen Caucasian male subjects consecutively attending the outpatient clinic of the Retina Medical Service at Bietti Foundation from 1 September 2018 to 15 December 2019 were enrolled. They were aged between 40 and 60 years and were newly diagnosed with an acute episode of unilateral idiopathic CSC, confirmed by fluorescein angiography and spectral-domain optical coherence tomography (SD-OCT) B-scan (Heidelberg Engineering, Heidelberg, Germany) (32–34). Furthermore, in order to exclude occult choroidal neovascularization, patients underwent to indocyanine green angiography (35).

Seventeen age-matched controls were recruited among Bietti Foundation employers/subjects accompanying patients to the visit without any ocular pathologies confirmed by the ophthalmological checkup and SD-OCT scan.

The exclusion criteria were: chronic/recurrent CSC (duration of visual symptoms for more than 12 weeks), and the choroidal neovascularization. Furthermore, uveitis history, optic disk edema, choroidal infiltrates were also excluded. None of the study participants had received in the previous 12 months any drugs (steroidal anti-inflammatory, immunosuppressive drug, and vasoactive/psychoactive treatments) that could have potentially affected their cortisol level.

The routine laboratory tests were all normal.

The same population was previously studied for the CSC-related involvement of the autonomic nervous system (ANS) activity by measuring salivary α-Amylase production in different day times (19).

### Spectral-domain optical coherence tomography scan protocol

The SD OCT images were acquired with Spectralis OCT (Heidelberg Engineering, Heidelberg Germany). The macular area was analyzed with 25 B raster scans centered on the fovea (the volume scan was 20° × 20°), and the EDI-OCT tool was activated to enhance the visualization of the choroid-sclera boundary. The value of the subfoveal choroidal thickness (FCT) was measured at the scan passing through the fovea, considered as the vertical space between the outer margin of the RPE and the choroid-sclera boundary. To estimate the interrater and intrarater observer changeability for the FCT measurement, all images were examined by two senior graders (ophthalmologists: EC and FS), and the intraclass correlation coefficients (ICCs) were computed for statistical analyses. Values of ICC < 3.5% were considered poor agreement. In the present study, the ICC was 0.87 for intrarater agreement and 0.89 for interrater agreement.

For the choroidal analysis, the CVI was calculated as follows (29): an EDI-OCT foveal scan was examined by using ImageJ software version 1.50 (National Institutes of Health, Bethesda, MD, USA), as previously described (36, 37). To ascertain the total choroidal area (TCA), a polygon tool was used to define the total subfoveal scan and added to the region of interest (ROI) manager. The Niblack automatic local threshold was used to binarize the image, which was previously converted into an 8-bit image and then transformed into red, green, and blue images. To compute the luminal choroidal area (LCA), the color threshold tool was used to measure the dark pixels, which were consequently added up with the ROI manager; white pixels were considered the stromal choroidal area (SCA), and the ratio between LCA and TCA was used to calculate the CVI (37).

### Spectral-domain optical coherence tomography angiography scan protocol

To study the choriocapillaris (CC), the study eye of each patient underwent SS-OCTA imaging with the PLEX Elite 9000 tool, and a 3 × 3 mm scan pattern centered on the fovea was used. Images showing detachment of the pigmented epithelium were not included in the study.

In the analysis, the total flow signal void area of the CC was considered. This area shows the total area of the CC vascular failures as a ratio of each examined area (38, 39). Additionally, superficial capillary plexus (SCP) and CC face OCTA, automatically provided by SS-OCTA, were evaluated using ImageJ software version 1.50 (National Institutes of Health, Bethesda, MD, USA). The SCP portion was segmented between the internal limiting membrane and the inner plexiform layer. The CC slab of 15 mm was considered starting from 16 mm below the RPE/Bruch’s membrane complex. All images were evaluated to exclude segmentation errors before processing.

The “Max Entropy” threshold was used when opening the enface SCP image to show only the greater superficial retinal vessels, which might cause shadowing and artifacts (27). The Phansalkar method was used to dichotomize the CC images, which were processed with the analyze particles tool to gather information on the total flow signal void area (40). Then, these two images were combined to exclude possibly confounding artifacts because of shadowing or projection, as previously shown (27, 38, 39). The images were processed with the “Analyze Particles” tool to quantify the total flow signal voids.

### Experimental procedure

Briefly, written informed consent was obtained from all participants. Furthermore, clinical and demographic characteristics were collected, and all subjects were taught how to collect their saliva at home using the Salivette sampling device (Sarstedt, Germany). They were asked to avoid eating, coffee, teeth brushing and any physical effort 30 min before each saliva collection (24, 41, 42).

Home diurnal saliva collection was planned on the sampling day upon waking (always between 07:00 and 08:00 h), 30 and 60 min after awakening. The day after, subjects returned the samples to the clinic, and the presence of CSC at the time of salivary collection was confirmed by a further SD-OCT scan. All participants were asked to text both available coauthors (FP, FS) at the planned collection time.

### Saliva collection and biomarker assay

As previously described in details saliva was collected using the Salivette sampling device (Sarstedt, Germany) (24, 41). Biomarker assays was obtained by commercially available kits (Demeditec-Diagnostic, Kiel, Germany) with the inter-assay coefficient of variation <10%, and the intra-assay coefficient of variation <7% (minimum detectable concentration of 0.5 ng/ml) (24, 41).

### Statistics

The statistical analyses and data visualization were performed by the SigmaPlot-11 software package (SxST.it, Italy). All quantitative variables were reported in the results as the mean and SE, unless otherwise specified. For each subject, raw biomarker data were used to estimate the Δ% increase in salivary CAR assessed by applying the formula CAR Δ% variation = [(cortisol level 30 min after awakening-awake cortisol level)/awake cortisol level] × 100.

The Kolmogorov–Smirnov test was applied prior to statistical analyses to check the normality of the distribution and the homogeneity of variance. Student’s *t-*test and the Mann–Whitney *U* test were applied for group comparisons. Pearson’s correlation test was used to examine the relationships between variables. The statistical significance was set at *P* < 0.05 (43).

## Results

### Demographic and clinical characteristics across the study population

All enrolled subjects were Caucasian men. Table 1 reports the demographic and clinical data of patients (*n* = 17) compared with those of the controls (*n* = 17). The control and CSC groups showed no differences in age or educational level. Furthermore, the two groups did not show statistically significant differences in body mass index or basal cardiovascular parameters. There were no smokers in the two groups, and alcohol consumption limited to meals was moderate, with less than 1 drink per day (44).

**TABLE 1:** Somatic and demographic characteristics of the study population.

	Control (*n* = 17)	CSC (*n* = 17)	Statistics	*P*-value
Age, years	50 (11)	48 (7)	*t* = −1.616	0.542
BMI, kg/m^2^	29.4 (5.4)	27.5 (7.3)	*U* = 09.000	0.228
Educational level, years	14.8 (2.9)	13.3 (3.1)	*U* = 101.500	0.113

Data are expressed as mean (SD). Statistics: Two-group comparisons were performed with Student’s *t*-test for continuous variables approximating a normal distribution or with Mann–Whitney *U*-test for continuous non-normally distributed variables. BMI, body mass index.

### Choroidal and choriocapillaris spectral-domain optical coherence tomography angiography image metrics in the study population

The comparison of imaging biomarkers between the study eye and control eye shows that eyes with CSC presented significantly higher FCT (control: 254 ± 13.1 SD: 53.9; CSC: 394 ± 23.3 SD: 96.03; Mann–Whitney *U* test for continuous non-normally distributed variables: *U* = 29,000, *T* = 182,000, *P* < 0.001) and CVI values (control: 62.26 ± 0.5 SD: 2.28; CSC: 76.45 ± 0.8 SD: 3.45; Mann–Whitney *U* test for continuous non-normally distributed variables: *U* = 47,000, *T* = 200,000, *P* < 0.001) than healthy control eyes. The evaluation of OCTA images of the choriocapillaris revealed that the prevalence of the flow signal void area was higher in the CSC group than in the control group (control: 31.92 ± 1.88 SD: 7.77; CSC: 36.96 ± 1.60 SD: 6.60; Mann–Whitney *U* test for continuous non-normally distributed variables: *U* = 92,000, *T* = 245,000).

**FIGURE 1:**
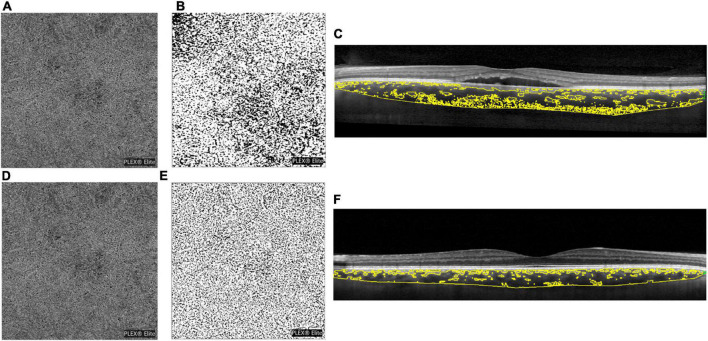
**(A)** Optical coherence tomography angiography (OCTA) scan of a central serous chorioretinopathy (CSC) eye after choriocapillaris segmentation and exportation from the PLEX Elite 900 device, showing a hyporeflective flow signal void area. **(B)** The choriocapillaris images binarized with the Phansalkar method for the quantification of flow signal voids by using ImageJ (public domain software). **(C)** Enhanced depth optical coherence tomography images highlighting the choroidal vascularity index (CVI). Image binarization was performed with the Niblack autolocal threshold with ImageJ (public domain software) to calculate the total, luminal, and stromal choroidal areas [total choroidal area (TCA), luminal choroidal area (LCA), and stromal choroidal areas (SCA)], and the CVI was calculated as the ratio between LCA and TCA. **(D–F)** Images in the healthy eye.

Figure 1 shows representative images of the CSC and healthy eyes, displaying hyporeflective flow signal void areas and the CVI.

### Salivary cortisol awake response delta percentage variation in the study population

**FIGURE 2:**
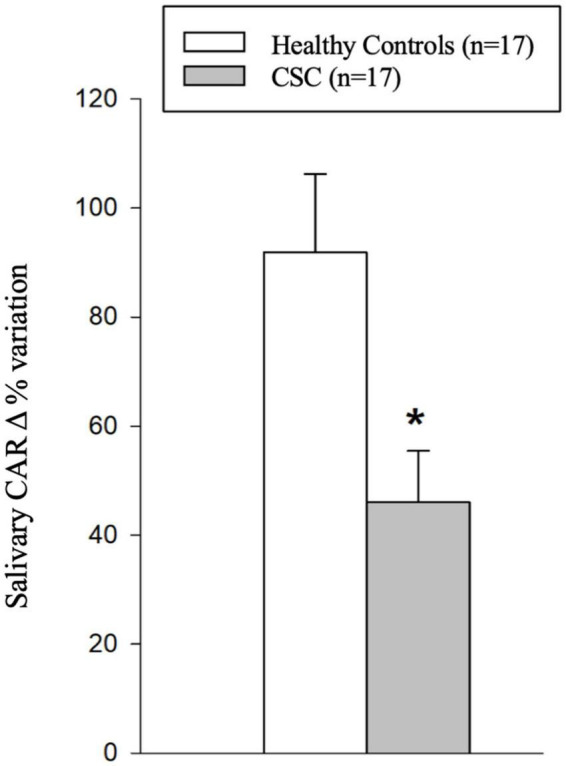
Histograms representing the salivary cortisol awake response (CAR) delta percentage (Δ%) variation in the study population. Statistics: * Student’s *t-*test for continuous normally distributed variables: *t* = 2.925; *p* = 0.006 vs. controls.

Figure 2 shows the CAR Δ% change reported as the percentage variation of salivary cortisol measured in both groups 30 min after awakening.

The raw data reveal the expected physiologically significant presence of CAR in the first half-hour after waking up in both the control (salivary cortisol at awakening: 3.6 ± 0.23 SD: 0.97 ng/ml; 30 min after awakening: 6.39 ± 0.34 SD: 1.42 ng/ml; Student’s *t*-test: *t* = −6.789 *p* < 0.001 vs. awakening; 60 min after awakening: 4.17 ± 0.25 SD: 1.06 ng/ml; ns vs. awakening) and CSC (salivary cortisol at awakening: 6.26 ± 0.53 SD: 2.179 ng/ml; 30 min after awakening: 8.81 ± 0.63 SD: 2.614 ng/ml; Mann–Whitney rank-sum test: *T* = −207.000 *p* = 0.002 vs. awakening; 60 min after awakening: 6.25 ± 0.58 SD: 2.40 ng/ml; ns vs. awakening). However, as depicted in Figure 2, data transformation in Δ% points out that subjects with CSC show, 30 min after awakening, a significantly lower increase in CAR Δ% variation (+46.0 ± 9.40; SD: 38.88) in comparison with controls (+79.8 ± 6.63; SD: 27.33; Student’s *t-*test for continuous normally distributed variables: *t* = 2.925; *p* = 0.006).

### Relationships between salivary cortisol awake response delta percentage variation and choroid-retinal metrics in the study population

**FIGURE 3:**
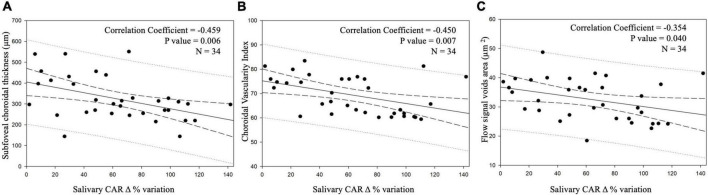
Scatterplots showing relationships between salivary cortisol awake response (CAR) delta percentage (Δ%) variation and the selected biomarkers of central serous chorioretinopathy (CSC) imaging in the study population: subfoveal choroidal thickness (FCT) **(A)**, choroidal vascularity index (CVI) **(B)**, and total flow signal void areas **(C)** in the study population. Continuous lines represent the best-fit linear regression; long dashed lines represent confidence bands; dotted lines represent prediction bands.

Figure 3 depicts scatterplots showing the statistically significant relationships between salivary CAR Δ% variation and FCT (Figure 3A), CVI (Figure 3B), and the flow signal void area (Figure 3C) in the study population.

## Discussion

In the present study, EDI-OCT scans followed by SS-OCTA showed that CSC subjects had increased FCT and CVI, as well as a higher distribution of total flow signal void areas in comparison with the matched controls, confirming and providing additional evidence that CSC may be caused by increased hydrostatic pressure in the choroid (45).

Hypothalamic-pituitary adrenal axis activity, assessed by measuring the trend of salivary cortisol production over the first hour after awakening, showed that the CAR Δ% variation was physiologically elevated 30 min after awaking in both the control and CSC groups. However, the CSC patients showed a remarkably blunted CAR Δ% variation in comparison with the controls, which might reflect CSC-related dysregulation of HPA axis activity. A key result of the present study is that a statistically significant relationship was selectively found between the salivary CAR Δ% variation and FCT, CVI, and flow signal void area in the study participants.

Overall, the present study confirms that CSC involves a generalized overproduction of salivary cortisol compared controls at awakening and 30 and 60 min later (6–8). Hypercortisolism has been related with hypertension and/or obesity (46, 47). However, the confounding effects of these comorbidities were avoided since the subjects enrolled in the present study were not obese (BMI < 30 kg/m^2^) or hypertensive (47).

Cortisol awake response is a useful index of the major neuroendocrine stress response system, and it was found to be altered among people with post-traumatic stress, fatigue symptoms, burnout, or exhaustion (26, 48). Furthermore, altered CAR has been described in psychiatric disorders (49) and several stress-related diseases (50–52). CAR was not detectable in the OSA population, (52) while it was restored after 3 and 6 months of continuous positive airway pressure (CPAP) therapy (53).

The data reported here are part of a larger study that previously showed that in acute CSC, the production of cortisol and the scores on the Daily Hassles and Stress Scale were higher in CSC subjects than in controls (5), resulting in allostatic overload and leading to erratic neuroendocrine responses (54). Thus, CSC subjects might be experiencing a pathogenetic process with a high level of stress hormone production in association with elevated subjective stress perceptions induced by daily hassles.

As a whole, by measuring the magnitude of the CAR, we observed in the present study an imbalance in the functional chronobiology of the stress system in CSC patients at awakening, while diurnal rhythmicity was preserved (8). We believe that this should be included among the CSC stress-related features (5, 18, 19, 55). In fact, the magnitude of the morning production of salivary cortisol followed a distinct trend in the CSC group during the first half hour of waking up with a lower CAR Δ% variation in comparison with healthy subjects.

The ANS dysregulation was previously highlighted in CSC subjects showing an overall higher diurnal production of salivary a-AMY, representing a sympathetic “drive” playing a role in the stress-induced pathophysiology of CSC (19, 55).

As previously shown, the aforementioned anomalies affecting salivary cortisol production at awakening are consistent with HPA axis imbalance in CSC, very likely in association with elevated subjective stress perceptions induced by daily hassles (5). For this purpose, McEwen’s allostatic load model for stress (56) originally speculated that a flatter pattern of cortisol secretion produced in response to physio-pathological challenges reflects an alteration of HPA activity that protects the stress system from dysfunction (57).

A weakness of the study is the absence of follow up. However, a prolonged decline in vision may contribute to altered HPA axis activity; thus, careful follow-up of patients should be taken into consideration in future studies to expand the understanding of the relationship between stress-related psycho-neuroendocrine imbalance and CSC. Furthermore, future research will be necessary to confirm the present results in women and elderly subjects, and also to consider the day-to-day variability not measured in the present protocol (58, 59).

Stress hormones have been linked to health and wellbeing. There are, however, a few studies on how the imbalance of the HPA axis, in the absence of particularly stressful stimuli during everyday life, might represent a marker of a functional disease. With this regard, in clinical practice, an example occurs in Cushing’s syndrome, which is diagnosed by measuring an elevation in late-night salivary cortisol (nadir) (60). The present study suggests the possibility that measuring salivary CAR patterns could be a specific and sensitive method for the determination of HPA axis imbalance in individuals with CSC (61).

As a whole, we believe that further investigation will ascertain the mechanism by which the dysregulation of the HPA axis leads to CSC, and altered functional chronobiology during wakefulness should be included among some of the stress-related CSC features (19) somehow linked to the choroidal hyperpermeability seen in CSCs. Future mechanistic studies are clearly needed, as corroborated by the fact that the salivary CAR correlates with choroidal and choriocapillaris features.

Finally, a follow-up of the patients already affected and then healed from the CSC will clarify whether the healing process is associated with a normalization of the CAR. If so, these patients might have a lower risk for recurrence, providing ophthalmologists with a new subclinical indicator of the disease and a possible therapeutic target (62).

## Data availability statement

The raw data supporting the conclusions of this article will be made available by the authors, without undue reservation.

## Ethics statement

Approval for this study was obtained from the Central Ethics Committee for Lazio, Italy (protocol no. 4327/18 April 2018). The patients/participants provided their written informed consent to participate in this study.

## Author contributions

FS, FP, and MP: conceptualization, project administration, and writing—review and editing. FS, FP, EC, and MP: data curation, investigation, methodology, and validation. MP: resources. FS and FP: software and writing—original draft. FP: supervision. All authors have read and approved the final manuscript.
